# Exploring the landscape of Lipid Nanoparticles (LNPs): A comprehensive review of LNPs types and biological sources of lipids

**DOI:** 10.1016/j.ijpx.2024.100305

**Published:** 2024-11-18

**Authors:** Alanood S. Alfutaimani, Nouf K. Alharbi, Amirah S. Alahmari, Almaha A. Alqabbani, Abdulaziz M. Aldayel

**Affiliations:** a Nanomedicine Department, King Abdullah International Medical Research Center, King Abdulaziz Medical City, Riyadh 11426, Saudi Arabia; b King Saud bin Abdulaziz University for Health Sciences (KSAU-HS), King Abdulaziz Medical City (KAMC), Riyadh 11426, Saudi Arabia; c Department of Biology, College of Science, Princess Nourah Bint Abdulrahman University (PNU), P.O Box 84428, Riyadh 11671, Saudi Arabia; d The Ear, Nose, and Throat (ENT) Department at King Salman Hospital, Riyadh 12769, Saudi Arabia

**Keywords:** Lipid nanoparticles, Lipids, Biological sources, Biotechnology, Drug delivery

## Abstract

Lipid nanoparticles (LNPs) have emerged as promising carriers for delivering therapeutic agents, including mRNA-based immunotherapies, in various biomedical applications. The use of LNPs allows for efficient delivery of drugs, resulting in enhanced targeted delivery to specific tissues or cells. These LNPs can be categorized into several types, including liposomes, solid lipid nanoparticles, nanostructured lipid carriers, and lipid-polymer hybrid nanoparticles. The preparation of LNPs involves the manipulation of their structural, dimensional, compositional, and physical characteristics via the use of different methods in the industry. Lipids used to construct LNPs can also be derived from various biological sources, such as natural lipids extracted from plants, animals, or microorganisms. This review dives into the different types of LNPs and their preparation methods. More importantly, it discusses all possible biological sources that are known to supply lipids for the creation of LNPs. Natural lipid reservoirs have surfaced as promising sources for generating LNPs. The use of LNPs in drug delivery is expected to increase significantly in the coming years. Herein, we suggest some environmentally friendly and biocompatible sources that can produce lipids for future LNPs production.

## Graphical abstract

**Figure:**
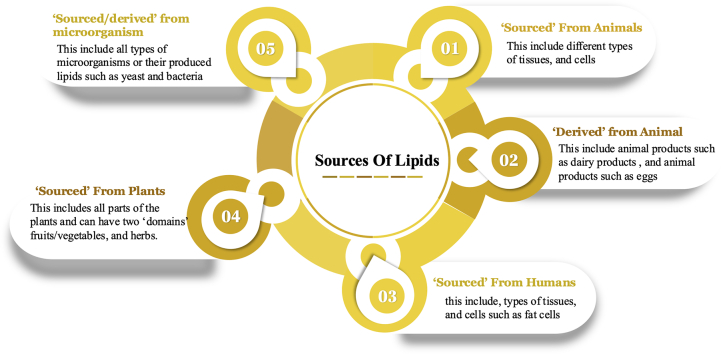

## 1 Introduction

**Fig. 1:**
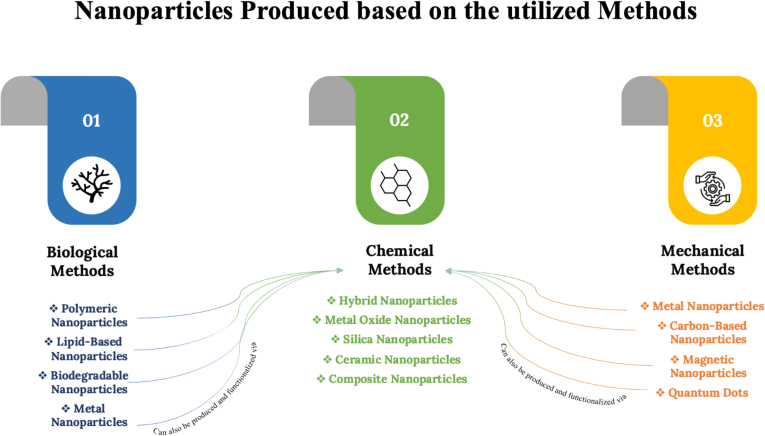
Different types of nanoparticles categorized by a production method. Nanoparticles are categorized based on the most common method of production used in the literature, and more than one production method may be applied to produce the same type of nanoparticles. The arrows indicate that all types of nanoparticles can be produced (formulated) functionalized (modification of the surface of the particle) using chemical methods. This figure visualizes the nanoparticle types and its production methods.

Nanotechnology is a branch of science that focuses on the manipulation of matter at a nanometer scale, involving the physical, chemical, and biological attributes of materials (Leon et al., 2020; Bayda et al., 2019). In particular, their role in the field of nanomedicine motivated the development of cutting-edge products such as therapeutics, vaccines, diagnostics, and preventative products (Subramaniam et al., 2010). Nanoparticles have been used to enhance the therapeutic and diagnostic capacity of several diseases, such as cancer (Fu et al., 2018). There are two main approaches to creating nanoparticles within the nanomedicine field. One of which is the top-down method, which begins with bulk material and meticulously chisels it down to the desired nanoparticle size (Mehnert and Mäder, 2001). This approach involves multiple strategies, such as physical methods which include lithography and milling, as well as chemical methods such as etching and decomposition (Ganesan and Narayanasamy, 2017). On the other hand, the bottom-up method is based on the power of nucleation, which involves controlled precipitation or crystallization. The principle of this approach is that atoms or molecules aggregate together forming a stable nucleus, which then acts as a growth platform for the formation of nanoparticles (Mehnert and Mäder, 2001; Abid et al., 2022; Chan and Kwok, 2011). This process allows for the versatility of the nanoparticle composition and structure (Chan and Kwok, 2011). The type of nanoparticles produced varies depending on the method used and competition (Arole and Munde, 2014). Fig. 1 shows an overview of different types of nanoparticles categorized by production method, wherein each production method can be applied to various raw materials.

Nanoparticles are currently used in drug delivery to overcome some challenges such as limited bioavailability, fast metabolism, toxicity, and rapid plasma level fluctuations (Mehnert and Mäder, 2001). Lipid nanoparticles (LNPs) combine the advantages of polymeric nanoparticles, liposomes, and microemulsions (Duan et al., 2020). LNPs are more pharmacokinetically acceptable, form inclusion complexes, and have improved stability patterns (Duan et al., 2020). Manufacturing LNPs utilizes triglycerides and fatty acids among other components (Jacob et al., 2022). Those lipids can be naturally produced by plants, animals and microorganisms (Koreti et al., 2022).

Engineered nanoparticles (NPs), such as liposomes, dendrimers, and polymeric NPs, have been shown to enhance the precision of drug delivery systems (DDS) by facilitating targeted delivery to specific cellular or tissue sites, such as inflamed or cancer tissue (Aldayel et al., 2023). The nanoparticles' size and shape are crucial parameters that influence their biological interactions and are considered when engineering the nanoparticles. In addition, functional coatings can be added to the surface of nanoparticles to enhance their targeted effect (Aldayel et al., 2016). For instance, NPs can be functionalized with ligands that recognize and bind to specific cellular receptors, ensuring site-specific drug release (Niu et al., 2014). This targeted approach minimizes systemic side effects and maximizes therapeutic efficacy. Other modifications to the surface of NPs prolong the presence of NPs in the bloodstream and improve their accumulation in target tissues through the enhanced permeability and retention (EPR) effect, which is particularly beneficial in targeting tumors and inflamed tissues with leaky vasculature (O'Mary et al., 2017). The composition of LNPs is also manipulated to increase the ability to hold and protect certain substances, such as nucleic acids, and facilitate their targeted delivery to specific cells for gene therapy applications (Aldayel et al., 2016). These strategies have the potential for NPs to be used in gene therapy, offering therapeutic solutions for genetic disorders (Hald Albertsen et al., 2022).

## 2 Lipid nanoparticles

Lipid nanoparticles (LNPs) are nano-delivery systems made of lipids that can carry drugs or genetic material into the body (Duan et al., 2020). LNPs have gained interest in the field of biotechnology and have been approved by the Food and Drug Administration (FDA) for drug and vaccine delivery (Shah et al., 2014). Their unique phospholipid bilayer structure offers a multifaceted solution due to their ability to encapsulate drugs within their aqueous core or lipid bilayers (Basu and Basu, 2008). This provides precise control over drug release, enhancing therapeutic efficacy and reducing potential side effects by ensuring drugs reach their intended targets in a controlled manner (Jacob et al., 2022). Moreover, their limited biotoxicity makes them ideal for medical applications (Basu and Basu, 2008). Additionally, the ease of large-scale production and sterilization of lipid-based nanoparticles is favored for pharmaceutical manufacturing (Shah et al., 2014). Their well-documented scalability measures remove most of the concerns with regards to manufacturing obstacles, thereby ensures an easier transition from lab-scale formulations to large-scale commercial production (Yeo, 2013).

**Fig. 2:**
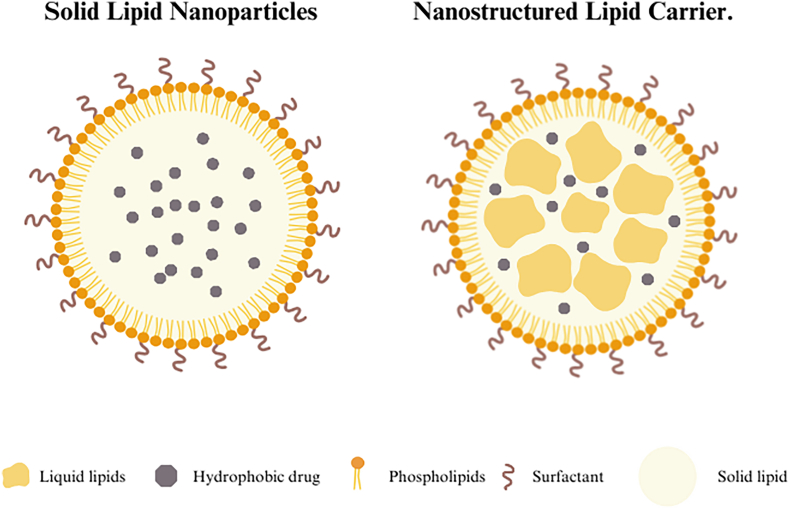
A simple visual aid to view Solid Lipid Nanoparticles (SLN) and Nanostructured Lipid Carriers (NLC) structure. LNPs can be categorized based on the type of lipids used and their configuration. For example, by integrating liquid lipids, as demonstrated in NLS, during the formulation the structure changes which in turn affects it's characteristics and type.

The structure and composition of LNPs vary and depend on the type of lipids and methods used for synthesis. For instance, Solid Lipid Nanoparticles (SLNs) are typically composed of lipid(s) that are solid at room temperature (Pink et al., 2019). Their structure has three different morphologies based on the location of the incorporated drug molecule: the drug-enriched shell model, the drug-enriched core model, and the homogenous matrix model (Geszke-Moritz and Moritz, 2016). Nanostructured Lipid Carriers (NLCs) are a development from SLNs designed to avoid problems with lipid crystallinity and polymorphism (Naseri et al., 2015). They are made using a binary mixture of two spatially different solid lipid matrices: a solid lipid and a liquid lipid (or oil) (Müller et al., 2016). Their structure is influenced by the organization of lipids and drugs in the particles, leading to a variety of structural models (Fig. 2) (Geszke-Moritz and Moritz, 2016; Müller et al., 2016). Lipid-drug conjugates (LDCs) involve the chemical conjugation of drugs with lipids (Irby et al., 2017). On the other hand, Polymer-Lipid Hybrid Nanoparticles (PLNs) are formed by combining polymers with lipids to create a polymer-lipid core. PLNs combine the properties of both lipids and polymers (Tahir et al., 2020).

The utilization of lipid nanoparticles presents a compelling alternative to conventional colloidal carriers. Each variant stated above addresses specific challenges associated with drug delivery, stability, and bioavailability, providing a versatile toolkit for pharmaceutical applications (Dhiman et al., 2021). Liposomes have been a powerful tool in medicine for over 50 years, capable of encapsulating and delivering therapeutics controllably to specific locations within the body for treating a variety of diseases (Tenchov et al., 2021). In 2008, SLNs transitioned from focusing primarily on basic Research and development (R&D) to exploring clinical applications (Zhou et al., 2019). More recently, there has been a promising approach to further personalizing and developing LNPs through the use of quantitative structure-activity relationship (QSAR) methods and artificial intelligence (AI) to predict LNP interactions with cells (Pozzi and Caracciolo, 2023).

Currently, lipid nanoparticles, including SLNs, NLCs, LDCs, and Polymer-Lipid Hybrid Nanoparticles (PLNs) are all currently in development at a pre-clinical, clinical and commercial level for drug and vaccine delivery.

### 2.1 Types of lipid nanoparticles

#### 2.1.1 Solid Lipid Nanoparticles (SLNs)

Solid lipid nanoparticles (SLNs) comprise lipids forming a core and surface stabilizers like phospholipids or bile salts (Naseri et al., 2015). Originally, SLNs were perceived as tiny, round particles made of solid lipids, but newer findings reveal they can also have disc-like or flat shapes (Scioli Montoto et al., 2020). The surface charge of these LNPs is influenced by the materials used in their making and the surrounding pH (Qushawy and Nasr, 2019). Both play a crucial role in the interaction of nanoparticles with their biological environment and their electrostatic interaction with bioactive compounds (Qushawy and Nasr, 2019). The complex nature of lipid modifications, such as the presence of various lipid forms (α, β, or β') and their interactions with emulsifiers, impacts the physical properties of SLNs such as particle size, which is particularly significant (Mehnert and Mäder, 2001).

SLNs are used in drug delivery due to their excellent bio-compatibility and small size, which enhance drug bioavailability and distribution (Mishra et al., 2018). Other advantages, such as the stabilization of hydrophobic drugs in biological systems and sustained drug release, are known to make this the ideal delivery system for hydrophobic drugs (Bayón-Cordero et al., 2019; Aldayel et al., 2018). However, potential burst release during storage and limited loading capacity for hydrophilic drugs, polymorphic transitions, and particle size growth over time are some of the reported concerns for SLNs (Ghasemiyeh and Mohammadi-Samani, 2018). Several methods and technologies are currently used in the industry to limit these concerns and make SLNs a viable drug delivery system for many applications. In addition, research has shown different ways to mitigate the inflammatory toxicities of nanoparticles by changing the formulation ratio of lipid nanoparticles and glucocorticoids (O'Mary et al., 2019).

#### 2.1.2 Liposomes

Since being discovered in the 1960s, liposomes have become a significant drug delivery method due to their biocompatibility, stability, simple synthesis, high drug loading, and safe ingredients (Nsairat et al., 2022). Liposomes are phospholipid-based drug carriers that self-assemble into vesicles, which can form a single unilamellar bilayer or multiple concentric multilamellar bilayers encasing an inner aqueous space. The inner aqueous space can vary in size from 30 nm to micrometers, with a phospholipid bilayer thickness of around 4–5 nm (Liu et al., 2022). Structurally, liposomes are spherical vesicles with a lipophilic bilayer between two hydrophilic layers, this composition offers versatility and benefits in drug delivery (Shah et al., 2014). Their vesicles are categorized by size and layers into small unilamellar vesicles (SUVs), large unilamellar vesicles (LUVs), multilamellar vesicles (MLVs), and multivesicular vesicles (MVVs). Their structure can vary, ranging from single-layered to onion-like or concentric spheres that contain smaller vesicles (Nsairat et al., 2022). The preparation and characterization of liposomes involve various techniques, including mechanical methods, solvent dispersion, and supercritical fluid technology, to control their size, encapsulation efficiency, and stability (Ahmed et al., 2019).

Their charge and structure significantly impact their interaction with the immune system, especially in vaccination and immunotherapy, as it has been shown in some studies that positively charged liposomes induce more effective antigen-specific cellular immune responses than negatively charged or neutral ones (Zahednezhad et al., 2019). Both the positive charge of liposomes and the chemical nature of the lipids used in their formulation play critical roles in determining their toxicity profiles. Cationic lipids, in particular, can disrupt cell membranes and induce apoptosis (Du et al., 2024). This interplay between immune activation and cytotoxicity highlights the need for careful consideration in therapeutic applications. The characterization of liposomes is critical for pharmacokinetic studies in advancing liposomal drug delivery systems, as they enable the detailed analysis required to understand the behavior of liposomal drugs in the body (Filipczak et al., 2020). Liposomes exploit the enhanced permeability and retention (EPR) for targeted drug delivery, particularly for anticancer drugs, as tumors often have leaky vasculature, allowing liposomal drugs to accumulate in tumor tissues while restricting entry into healthy tissues (Liu et al., 2022). Modifications like PEGylation enhance this effect by inhibiting recognition of the reticuloendothelial system and increasing prolonging liposomes' circulation time, which enhances the therapeutic efficacy of the drug (Sercombe et al., 2015). Presently, over 15 Liposome -based therapeutic products have received clinical approval, with numerous others in various stages of clinical evaluation. These LP-based drug formulations are designed for treating diverse conditions such as various cancers, and fungal, viral, and bacterial infections (El-Hammadi and Arias, 2019).

#### 2.1.3 Nanostructured Lipid Carriers (NLCs)

Around seven years after the introduction of solid lipid nanoparticles (SLN), a newer iteration known as nanostructured lipid carriers (NLCs) emerged and was seen as an upgraded version of SLN because they integrate small amounts of liquid lipids (oils) into their structure at room temperature, causing structural changes in the matrix (Gordillo-Galeano and Mora-Huertas, 2018). This adaptation addressed a notable observation: over time, the crystalline structure of SLNs tends to mature, leading to the potential release of the enclosed drug into the surrounding medium (Ganesan and Narayanasamy, 2017). Nanostructured Lipid Carriers (NLCs), the mixture of solid and liquid lipids forms a less ordered matrix that's less prone to crystallization as liquid lipids disrupt the crystalline structure of the solid lipids, resulting in an imperfect crystal lattice (Khan et al., 2023).

This is achieved by using various lipid types, including triglycerides, partial glycerides, fatty acids, steroids, and waxes, which have been employed in NLCs formulations (Elmowafy and Al-Sanea, 2021). The NLC classifications, which include three main domains, amorphous, imperfect, and multiple types, vary in their drug delivery behavior (Akbari et al., 2022). Amorphous NLCs, also known as NLC type II, are mixed in a way that prevents crystallization, resulting in a solid but non-crystalline lipid matrix that minimizes drug release by maintaining the lipid matrix in an amorphous state (Journal of Developing Drugs, 2018). This structureless type of NLCs prevents crystalline structured matrix development seen with the β polymorph of solid lipids, thus enhancing drug loading capacity and stability (Salvi and Pawar, 2019). The drugs incorporated within these particles are found in their amorphous form, indicating that the drug molecules are arranged in a disordered, non-crystalline structure, and are maintained even after undergoing temperature-induced transformations, such as melting and re-crystallization during storage (Tetyczka et al., 2019). On the other hand, imperfect NLCs (type I) are a type of NLCS characterized by their internal structure, which includes imperfections or irregularities. These imperfections can be due to the mixture of solid and liquid lipids that do not form a perfectly ordered crystalline structure (Apostolou et al., 2021). The presence of liquid lipids disrupts the perfect crystalline structure of the solid lipids, leading to a less ordered matrix that can accommodate more drug molecules in an amorphous or less ordered state (Tamjidi et al., 2013). It also allows for the loading of various hydrophilic and hydrophobic substances (Mall et al., 2024).

#### 2.1.4 Polymer-Lipid Hybrid Nanoparticles (PLNs)

Polymeric Lipid Hybrid Nanoparticles (PLNs) are stable with biocompatible core-shell nanoparticles that effectively combine the properties of liposomes and polymeric nanoparticles (Mall et al., 2024). These nanocarriers are increasingly utilized in biomedical applications like drug delivery and imaging due to their efficient medication distribution, superior stability, and easy preparation (Sivadasan et al., 2021). PLNs are a breakthrough in drug delivery, especially for cancer treatment, due to their controlled drug release, increased drug loading capacity, and extended circulation time (Parveen et al., 2023). PLNs are categorized based on structural arrangements, which include various configurations of lipids and polymers and come in various forms (Dave et al., 2019). One common structure is the polymer core lipid shell, which has a single or bilayer of lipids coating a polymer partial core, This design provides a lipid layer surrounding a hollow polymer core (Wu, 2016). The other most known form, monolithic structured PLN, integrates both lipids and polymers into a single, homogeneous matrix which ensures a stable and controlled environment for drug encapsulation and release, making it suitable for various biomedical applications including targeted drug delivery and cancer therapy (Persano et al., 2021). By combining the advantages of both liposomes and polymers, PLNs are poised to significantly enhance therapeutic efficacy in various medical fields.

### 2.2 The recent application of LNPs in nanomedicine

Over the last 5 years, there have been a multitude of approaches utilizing SLNs. One paper discussed the recent patents and research articles published in this field, they observed advancements in the development of compositions and formulations that improved therapeutic outcomes, cosmetic applications, and nutraceutical applications (Paliwal et al., 2020). One of which is the Quality by Design (QbD) approach, which enhances the manufacturing of lipid nanoparticles and nanoemulsions for drug delivery, ensuring pharmaceutical product quality and safety (Cunha et al., 2020). Another approach was to utilize mathematical models and statistical tests to identify and control critical parameters in lipid-based nanosystems, such as SLNs, NLCs, and nanoemulsions (González-Fernández et al., 2021). This systematic approach helps in achieving the desired product quality and consistency in performance by providing better control over various formulation variables such as lipid concentrations, surfactant concentrations, and the pressure of high-pressure homogenizers (Gurumukhi and Bari, 2022). LNPs are used in the delivery of nucleic acids for gene therapy, cancer immunotherapy, and the rapid development of mRNA vaccines, notably against COVID-19, by engineering them to protect genetic material from degradation, enhance cellular uptake, and ensure targeted delivery to disease sites (Zhang et al., 2022). More recently, researchers have been interested in merging different types of LNPs with other delivery systems. For example, a group of researchers designed cell-platelet hybrid membranes to camouflage liquid crystalline lipid nanoparticles for drug delivery. This system is camouflaged with a hybrid membrane which combines features from both cancer cell membranes and platelet (PLT) membranes to leverage the long circulation time. Thus endowing the nanoparticles with the combined advantages of both types of membranes (Li et al., 2021).

Folate-targeted lipid-polymer hybrid nanoparticles (FLPHNPs) are another example designed for the targeted delivery of therapeutic agents. It is composed of a lipid polymer hybrid structure with a “folate-targeted” aspect such as folate (vitamin B9) or folate derivatives, which are used to specifically target cancer cells (Khan et al., 2020). Another paper evaluated the encapsulation and delivery of anastrozole (ANS) for breast cancer treatment (Massadeh et al., 2020). They found through fluorescence-activated cell sorting (FACS) analysis and Hoechst staining that ANS-PLNPs induce similar apoptotic effects and DNA damage in MCF-7 breast cancer cells as free ANS. This suggests that the encapsulated form of ANS retains its anticancer activity while potentially offering benefits such as targeted delivery and reduced side effects (Massadeh et al., 2020). Another study targeting breast cancer demonstrated that gemcitabine-loaded lipid-polymer hybrid nanoparticles (LPHNs) exhibited superior anti-tumor efficacy and improved pharmacokinetic profiles in breast cancer treatment compared to the commercial product Gemko (Yalcin et al., 2020). All examples highlight the potential of LNPs as an effective drug delivery system (Yalcin et al., 2020; Khan et al., 2021a). The attempts to ‘merge’ raw materials to produce different types of LNPs have been increasing in the literature. One study synthesized nanoporous silicon-stabilized hybrid lipid nanoparticles that act as delivery systems for the flavonoid quercetin to efficiently transport and release quercetin, a beneficial compound known for its protective effects on plants under stress conditions (Guerriero et al., 2023). Another approach was to combine nanostructured lipid carriers and immunotherapies for targeted drug delivery in cancer therapy. The combination approach enhanced the efficacy and selectivity of chemotherapy drugs by targeting specific receptors like VEGFR, EGFR, and HER2 (Marques et al., 2023).

One study utilized the interactions between sumac tannin and liposomes, which led to the development of hybrid liposomes with strong antibacterial activity against Gram-positive bacteria. This approach enhanced the thermodynamic properties, increased membrane rigidity, and formed lipid nanodomains, ultimately enhancing antibacterial efficacy (Sekowski et al., 2023). Manipulating the properties of these LNPs by incorporating F-L319 enhances mRNA delivery and increases efficiency both in vitro and in vivo compared to their non-fluorinated counterparts (Huo et al., 2023). The hybrid LNPs containing a combination of F-L319 and L319 showed improved cellular uptake, endosomal escape, and overall mRNA expression, making them a promising strategy for targeted drug delivery (Huo et al., 2023). Another study focused on optimizing zein-based hybrid lipid nanoparticles for targeted drug delivery against prostate cancer cells (Maeyouf et al., 2023). The co-encapsulation of docetaxel and employing transferrin for cell targeting enhanced cellular uptake and anti-proliferative efficacy (Maeyouf et al., 2023). A new approach that exhibits promising potential in cancer therapy is hybrid magnetic lipid-based nanoparticles. By combining hyperthermia, chemotherapy, and photodynamic therapy it offers a unique multifaceted approach that enables targeted therapy. The external magnetic fields guide the nanoparticles to tumor sites, where exposure to an alternating magnetic field induces hyperthermia, enhancing the direct killing of tumor cells (Luiz et al., 2023).

### 2.3 Structure and composition of Lipid Nanoparticles (LNPs)

The structure and composition of LNPs vary depending on the molecule they are designed to deliver. In this section, we focus specifically on the structure and composition of LNPs used for mRNA delivery, to better interpret how these characteristics change. The composition of lipids in the lipid nanoparticle formulation typically used today includes ionizable lipid, phospholipid, cholesterol, and PEG-lipid in the approximate molar ratios of 50:10:38.5:1.5, respectively (Hald Albertsen et al., 2022). The structure and stability of LNPs are influenced by the specific lipid components used in their formulation. For example, mRNA lipid nanoparticles (mRNA-LNPs) that integrate two different lipids were observed for its stability, as these cholesterol-rich liposomes have reduced protein association and slowed its clearance from the circulation (Kawaguchi et al., 2023).

Ionizable lipids are key for enabling LNPs to encapsulate nucleic acids efficiently and facilitate their endosomal escape into the cytoplasm, due to their ability to form cone-shaped ion pairs with anionic endosomal phospholipids upon protonation in the acidic endosome (Han et al., 2021). This phenomenon is known to facilitate endosomal escape and cargo release into the cytosol, which is crucial for the successful delivery of RNA therapeutics and vaccines (Han et al., 2021). The ionized nature of the lipid can play a significant role in the properties of synthesized LNPs.

One study concluded the importance of minimizing undesirable systemic off-target expression of mRNA-LNP vaccines (Carrasco et al., 2021). They reported that highly negatively charged LNP formulations can lead to higher off-target systemic mRNA expression in the liver after intramuscular administration (Carrasco et al., 2021). This is primarily due to pKa differences between the ionizable lipid and the LNP, influenced by proton solvation energy variations (Carrasco et al., 2021). Another example is the inclusion of 1,2-dioleoyl-sn-glycero-3-phosphoethanolamine (DOPE) in the composition of LNPs, which showed higher transfection efficiency in vitro and in vivo (Young et al., 2024). The immunogenicity of LNPs can be influenced by the choice of lipid components. For instance, cholesterol is used as a helper lipid for LNPs stability and may also suppress unwanted protein binding to LNPs. Studies have shown that the structure of the lipid part, including the saturation of lipid hydrocarbon chains and the type of lipid anchor (e.g., PE vs. diglyceride vs. sterol), affects the immune-mediated adverse effects (Tenchov et al., 2023). It is reported that ionizable lipids may boost immune responses by increasing IL-6 production and inflammasome activation, such as the incorporation of phosphatidylserine (PS), which enhances transfection efficiency and protein expression in secondary lymphoid organs (Chen and Blakney, 2024).

Lipid nanoparticle formulation is a tool to innovate different types via manipulating the composition, structure and formulation method. Allowing for a more personalized approach to drug delivery and better efficacy.

### 2.4 Methods of preparation

The fabrication of lipid nanoparticles involves the manipulation of their structural, dimensional, compositional, and physical characteristics (Roces et al., 2020). This is governed by the raw materials (lipid type and source) and preparation conditions, with two main approaches: low energy and high energy (Roces et al., 2020). The low-energy approach, also known as spontaneous formation, utilizes the power of self-assembly to construct LNPs within a surfactant oil in-water (SOW) system (McClements, 2013). It capitalizes on various phenomena, including temperature fluctuations, emulsion inversion points, inversion composition phases, and spontaneous emulsification (McClements, 2013). All of these exploit the inherent tendency of molecules to organize themselves into stable structures, resulting in the formation of LNPs with defined characteristics (McClements, 2013). During the use of low energy methods It's important to consider the parameters of formulation as they can affect the overall characteristics of the LNP's (Kamanzi et al., 2024). Furthermore, the transition that occurs during self-assembly from disordered state (residual entropy) of lipids to a structured one (formulation of LNP's) reduces the degrees of freedom for the lipid molecules and changes the dynamics of how materials move within these structures (Inguva et al., 2024).

High-energy approach uses mechanical homogenization to generate intense disruptive forces that integrate the oil phase, leading to the formation of lipid particles (Ganesan and Narayanasamy, 2017). Well-known instruments such as homogenizers and extruders are used to apply shear stress and turbulence to break down the oil phase into nanoscale droplets and facilitate the formation of LNPs (Ganesan and Narayanasamy, 2017). Regardless of the method, LNPs typically exhibit a simple core-shell structure composed of non-polar components that encapsulate the therapeutic cargo (Mehta et al., 2023). While the shell, which is formed from surface-active agents such as surfactants, provides stability and facilitates interaction with the biological environment (Mehta et al., 2023). The core-shell structure contributes to the efficacy and safety of LNPs by protecting the therapeutic drug from degradation and enabling targeted delivery to the desired site of action (McClements, 2013). The choice between the low-energy and high-energy approaches for LNP fabrication depends on several factors, such as particle size, the sensitivity of the active pharmaceutical ingredients (API), and the scalability of the process (Khairnar et al., 2022). For instance, the low-energy approach is often preferred for its simplicity and scalability, while the high-energy approach is for achieving smaller particle sizes or handling more viscous materials (Khairnar et al., 2022).

### 2.5 Different sources of lipids used to formulate LNPs

**Fig. 3:**
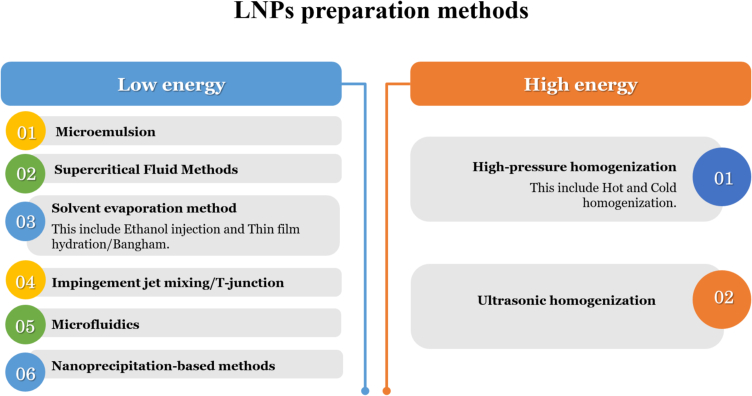
Different preparation methods of LNPs formation. Methods are usually classified based on the energy input to form LNPs. These methods can be broadly divided into two categories: low and high energy. Low energy techniques rely on spontaneous self-assembly of lipids in solution. These methods require minimal external energy and are often favored for their simplicity and scalability. In contrast, high energy methods involve the application of significant mechanical energy to form LNPs. These techniques typically involve breaking down lipid aggregates or emulsions into nanoparticles by applying intense shear forces or sonic waves.

**Fig. 4:**
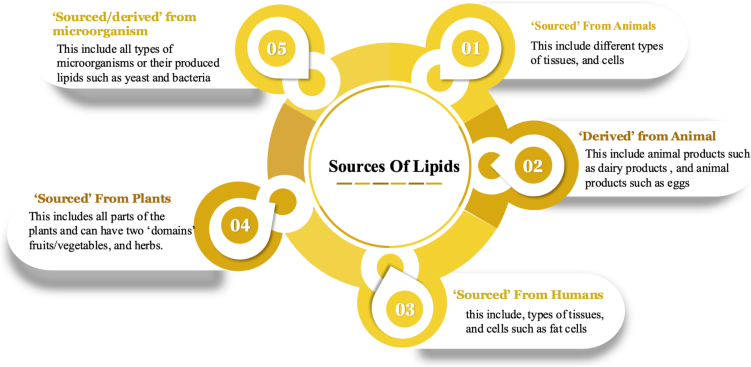
Bio-sources of lipids that can be utilized in LNPs formation. Lipids can be extracted from various biological sources. Through our research, we categorize them to 5 main sources, with the term ‘derived’ used for the product of the source while the term ‘sourced’ is used to refer to the use of the organism itself. Those sourced from plants and derived from microorganisms, animal and animal derived, and lastly human sourced. The sources listed provide lipids that are most commonly used in the formation of LNPs.

The selection and sourcing of lipids play a pivotal role in the formulation of lipid-based nanoparticles by influencing their composition, structure, and ultimately, their efficacy as drug delivery systems (Abumanhal-Masarweh et al., 2019). The choice of lipids not only determines the physical characteristics of LNPs but also significantly impacts their biocompatibility, stability, and drug-loading capacity. In particular, in terms of their saturation level, linkage type (ether or ester), and the presence of isoprenoid fatty acid tails (phytanyl lipids), they directly influence their thermal stability as well (Knetsch and Ubbink, 2024). Herein, we will discuss the documented sources of lipids utilized in LNPs formation (Fig. 3, Fig. 4).

#### 2.5.1 Animal source

##### 2.5.1.1 Marine animals

A study published in 1973 that compared lipid content in crude and purified extracts of marine organisms found higher lipid content in crude extracts like *Balanus balanoides* (26.3 % vs. 11.4 %) The types of lipids extracted include total lipids, which were estimated gravimetrically, and cholesterol, which was estimated using the Boehringer Test (Barnes and Blackstock, 1973). Indicating that 14.9 % of lipid content was lost during the purification process which can pose challenges when scaling up the process. In another study, researchers acquired lipids from the tissue of marine copepods and Artemia that include wax esters, triglycerides, and phospholipids, as well as several lipid classes in marine copepods and Artemia. The specific lipid classes quantified in the analysis include hydrocarbons, methyl esters, ketones, triglycerides, free fatty acids, sterols, partial glycerides, acetone-mobile polar lipids, and phospholipids (Gardner et al., 1985). One study explored lipid extracts' fatty acid composition from channel catfish muscle tissue and evaluated extraction methods where nine solvent systems were compared during the lipid extraction process, revealing similar recoveries for phospholipids and triacylglycerols with selected systems. The interaction time with the dichloromethane: methanol (9:1) system was crucial for effective phospholipid extraction (Erickson, 1993).

One study used fish tissues, specifically the liver tissues of hatchery-reared lake trout (*Salvelinus namaycush*) and whole body Atlantic herring samples, as well as white muscle from Atlantic bluefin tuna. They found that polar lipids comprised approximately 15 % of total lipids in liver tissues from hatchery-reared lake trout (*Salvelinus namaycush*), while carcass total lipids contained less than 1 % polar lipids (Logan and Lutcavage, 2008). The percentage of lipids removed is expected to increase for non-polar solvents as the percentage of polar lipids decreases (Logan and Lutcavage, 2008). It is important to note that the total lipid content extraction is not explicitly quantified in the provided citations, but rather the efficiency and impact of different extraction methods on lipid removal are discussed. In another study, a variety of fish and invertebrates, as well as commercial fish oil were added to ground commercial fish to produce mixtures with a range of estimated lipid contents. They weighed aliquots of a homogenous mixture of ground commercial fish (originally containing 2 % fat) and commercial fish oil to create mixtures with lipid contents ranging from an estimated 21 to 26 % (Iverson et al., 2001). Although the study aimed to compare lipid extraction methods, it did demonstrate the ability of these marine animals to become an economically viable source of lipids.

Another study targeted adult oysters, specifically the species *Crassostrea gigas*, and found only 7 % of the triacylglycerols were extracted from lyophilized samples compared with non-lyophilized “control” samples (Dunstan et al., 1993). There was no significant change in the amounts of polar lipids, total sterols, free fatty acids, or hydrocarbons observed in extracts from lyophilized samples relative to extracts from nonlyophilized samples (Dunstan et al., 1993). Generally, animals contained a high amount of unsaturated fatty acids, which in some cases comprised more than 65 % of the total fatty acids. Importantly, marine fish generally have a higher fraction of monounsaturated fatty acids (MUFA) and polyunsaturated fatty acids (PUFA) compared to freshwater fish (Table 1). Specifically, marine fish are noted to be good sources of omega-3 PUFA (PUFA-n3), with species such as *Nemipterus hexodon* (ornate threadfin bream), *Euthynnus affinis* (Eastern little tuna), and *Megalaspis cordyla* (hardtail scad) being highlighted for their PUFA-n3 content (*Fatty Acid Composition of Important Aquatic Animals in Southern Thailand*, 2011). Omega-3 PUFA lipids derived from marine animals have a recorded use in nanoemulsion (Gulotta et al., 2014). In addition, there has been a recorded use of marine lecithin to stabilize these nanoemulsions (Belhaj et al., 2010). Docosahexaenoic acid (DHA) (Valdes et al., 2019), and eicosapentaenoic acid (EPA) which are types of PUFA lipids are being used as lipid components of promising LNPs for drug and gene delivery (Lacatusu et al., 2013).

**Table 1:** Advantages and challenges of each source of nanoparticles.

Source	Advantages	Challenges
Marine Animals	- **High lipid content in some species:** Especially cold-water species. (Barnes and Blackstock, 1973). - **Rich in unsaturated fatty acids**: Including omega-3 PUFA (DHA, EPA) (Parrish, 1999). - **Divers lipid classes:** Including wax easters, triglycerides, and phospholipids (Parrish, 1999). - **Economic feasibility:** The commercial fishing industry produces large quantities of lipid-rich byproducts (*Fatty Acid Composition of Important Aquatic Animals in Southern Thailand*, 2011). - **Extraction methods:** Several effective methods such as enzymatic extraction, exist for retrieving these lipids from marine animals (Sardenne et al., 2019).	- **Variability:** Lipid composition can vary significantly based on factors such as species and diet (Logan and Lutcavage, 2008). - **Contaminant risks:** Marine animals, particularly those at higher trophic levels can accumulate harmful substances such as heavy metals (Patel et al., 2020). - **Processing complexity:** Often requires specialized techniques and equipment (Erickson, 1993). - **Allergenic potential:** Marine animals can trigger allergic reactions, which limits their use in some formulations (Lopata et al., 2010).
Insects and Bugs	- **Sustainability:** Insects like Black soldier fly (BSF) are emerging as a sustainable and environmentally friendly source of lipid. They can be grown on organic waste, making them a low- impact option (Franco et al., 2021a). - **Can be used as bioconverters:** Insects can efficiently convert waste products into lipids, helping to recycle nutrients and reduce waste **(****Franco et al., 2021a****)**.	- **Limited studies:** Despite growing interest, the data on lipid extraction from insects for biomedical applications is still limited (Coakley et al., 2019). - **Extraction optimization:** Current lipid extraction methods from insects require further refinement to maximize yield and efficiency (Coakley et al., 2019).
Mammals Waste	- **Diet manipulation:** The fatty acid composition of mammalian fats can be adjusted based on diet, making it possible to tailor lipid profiles for specific applications (Miller et al., 1986). - **Repurposing waste:** By utilizing fats from mammalian waste (such as tallow), industries can reduce waste and lower the cost of lipid production (Srinivasan et al., n.d.). - **Divers fatty acid profiles:** Mammalian fats offer a range of fatty acids, providing flexibility for different formulations **(****Veerkamp et al., 1962****)**.	- **Quality variability:** Depending on the source and processing methods, the consistency and quality of lipids can vary, making standardization difficult (Miller et al., 1986). - **Contaminant risks:** As with marine sources, there are risks of contamination in waste animal fats, including pathogens and chemical residues (Abdel-baky et al., 2020). - **Processing complexity**: Might need specialized techniques and equipment (Abdel-baky et al., 2020). - **Regulatory considerations**: Particularly with concerns over cross-species disease transmission (Abdel-baky et al., 2020).
Milk	- **Diverse lipid source:** Milk is naturally rich in lipids, including triglycerides phospholipids, and cholesterol (McManaman, 2009). - **Naturally structured as an oil-in-water emulsion:** Which is beneficial for nanoparticle formulations (McManaman, 2009). - **Biocompatibility:** Milk-derived lipids are generally well-tolerated by the human body, reducing risks of immune reactions or toxicity (Woods et al., 2022). - **Therapeutic benefits:** Components of milk lipids have been shown to possess anti-inflammatory and antioxidant properties, making them attractive for drug delivery systems (Woods et al., 2022).	- **Variable lipid composition:** The lipid composition of milk can change depending on the animal's species, diet, and health status. (Ibrahim and Gyawali, 2013). - **Processing difficulties:** Extracting and processing milk lipids can be technically challenging, particularly when trying to maintain bioactive properties **(****McManaman, 2009****)**. - **Allergenic potential: Milk** proteins are common allergens, particularly in sensitive populations (Aiuto et al., 2024). - **Environmental impact:** Dairy farming is associated with significant environmental concerns, including greenhouse gas emissions and water usage (Abdel-baky et al., 2020).
Egg yolk	- **Lipid concentration:** Egg yolks are one of the richest natural sources of lipids, particularly lecithin and phospholipids, which are useful for nanoparticle formulations. (Xiao et al., 2020). - **Emulsification**: Egg yolks are excellent emulsifiers, helping to stabilize lipid nanoparticles in aqueous solutions (Huopalahti et al., 2007). - **Diverse fatty acids:** They contain a variety of essential fatty acids, contributing to the structural diversity of formulations (Wood et al., 2021). - **Proven applications:** Egg yolk lipids have been widely used in pharmaceutical and cosmetic formulations (Wood et al., 2021).	- **High cholesterol content:** Egg yolk lipids are high in cholesterol, which may not be suitable for certain therapeutic applications (Rossi et al., 2013). - **Composition variability:** Like other animal-derived sources, the lipid composition can vary based on species and diet (Rossi et al., 2013). - **Processing challenges:** Separating and purifying egg yolk lipids can be labor-intensive and costly (Abdel-baky et al., 2020). - **Allergenic potential:** Egg proteins are common allergens, limiting their use in certain populations (Cansell, 2007). - **Regulatory considerations:** There are strict regulatory requirements when using egg-derived products, particularly in the pharmaceutical industry (Cansell, 2007).
Human	-**Biological relevance:** Human-derived lipids, such as those found in cell membranes, are physiologically relevant and mimic the natural environment of human cells (Kumar Srivastava et al., 2006). - **Specialized lipid classes:** Human cells contain lipid classes such as sphingomyelin and phosphatidylcholine, which have specific roles in cellular function and drug delivery systems (Skotland et al., 2019).	- **Ethical considerations:** The use of human-derived lipids raises significant ethical issues, particularly regarding tissue sourcing and consent. - **Heterogeneity:** Human-derived lipids can vary greatly between individuals complicating consistency in formulation (Skotland et al., 2019). - **Immunogenicity:** Even human-derived materials can provoke immune responses in some cases, necessitating careful safety testing (Clayton et al., 2008). - **Regulatory hurdles:** Regulatory approval for human-derived lipids can be difficult to obtain. - **Scalability:** Producing human-derived lipids on an industrial scale presents significant challenges (Skotland et al., 2019).
Plant source	- **Non-toxic and low immunogenicity:** Plant lipids are generally considered safe for human use, with low risk of adverse immune reactions (Sarvarian et al., 2022). - **Versatility:** These lipids can be chemically modified for various applications, including drug delivery (Tinnirello et al., 2023). - **Therapeutic potential:** Plant lipids such as phytosterols have therapeutic properties, including anti-inflammatory and anticancer effects (Zhu and He, 2023). - **Sustainable:** Plant lipids are renewable and environmentally friendly, making them a popular choice for nanoparticle formulations (Tinnirello et al., 2023).	-**Variability:** The composition of plant lipids can vary depending on the species growing conditions, and processing methods (Cao et al., 2019). - **Extraction challenges:** Efficiently extracting lipids from plants can be technically challenging and expensive (Cao et al., 2019). - **Allergenic potential:** Some plant lipids may cause allergic reactions in sensitive individuals **(****Streatfield, 2005****)**. - **Regulatory considerations:** As with other sources, there are regulatory hurdles to using plant-derived lipids in pharmaceuticals (Streatfield, 2005).
Microorganisms source	- **Cost-effective:** Microorganisms such as algae and yeast can produce lipids at a lower cost compared to traditional sources (Ghazani and Marangoni, 2022). - **Genetic modification:** These organisms can be engineered to produce lipids with specific characteristics or fatty acid profiles (Ghazani and Marangoni, 2022). - **Environmental sustainability:** Microbial lipid production is generally environmentally benign, using fewer resources than animal-derived sources (Kothri et al., 2020).	-**Technological and possessing challenges:** Scaling up microbial lipid production can be technically complex and expensive (Kothri et al., 2020). - **Regulatory and safety concerns:** There are safety concerns regarding the use of genetically modified microorganisms, and regulatory approval can be difficult (Patel et al., 2020). - **Competition:** Microbial sources face competition from more traditional lipid sources, making economic viability a concern (Patel et al., 2020). - **Risk of contamination:** Industrial-scale fermentation environments are prone to contamination by unwanted microbes. **(****Kothri et al., 2020****)**. - **Antibiotic Use and Resistance**: Widespread use of antibiotics can lead to the development of antibiotic-resistant strains (Demain and Sanchez, 2011).
Yeast	- **High lipid content:** Some yeast strains can accumulate up to 70 % of their dry weight as lipids, making them highly efficient lipid sources (Ratledge, 2004). - **Substrate Versatility:** Yeast can utilize a wide range of substrates, including industrial by-products, for lipid production (Wen et al., 2020). - **Customizable Profiles:** Yeast lipids can be tailored for specific applications through genetic and metabolic engineering (Fabiszewska et al., 2021). - **Industrial scale potential:** Yeast is robust and can be cultivated on an industrial scale, making it a promising source of lipid (Wen et al., 2020). - **Bio-renewable:** sustainable source of lipids (Naveira-Pazos et al., 2023).	- **Processing costs:** Scaling up yeast lipid production can be expensive, especially in terms of downstream processing (Naveira-Pazos et al., 2023). - **Regulatory considerations**: As with microorganisms, there are concerns over safety, particularly for genetically modified yeast (Kulagina et al., 2021). - **Competition:** There is competition from more traditional lipid sources (Naveira-Pazos et al., 2023). - **Production consistency:** Ensuring the production of high-purity and consistent-quality lipids can be a challenge (Kulagina et al., 2021). - **Allergenic potential:** Some people may have sensitivities or allergic reactions to yeast products (Kulagina et al., 2021).
Bacteria	- **High lipid accumulation:** Certain bacterial strains have a high capacity for lipid production, which can be advantageous for large-scale applications (Dong et al., 2016). - **Fast growth rates:** Bacteria can be cultivated rapidly, making them an efficient lipid source (Kumar et al., 2020). - **Genetic manipulability:** Bacteria are easily engineered, allowing for the production of custom lipid structures (Luo et al., 2022). - **Controlled production:** Bacteria can be grown under tightly controlled conditions, ensuring consistency in lipid output (Prema et al., 2015).	- **Lower lipid content:** compared to yeast (Koreti et al., 2022). - **Regulatory concerns:** As with other microbes, there are safety concerns with the use of genetically modified bacteria (Lancini and Demain, 2013). - **Complex processing:** Extracting and purifying lipids from bacteria can be challenging and requires specialized equipment (Prema et al., 2015). - **Consistency issues:** Ensuring uniform quality is a challenge in bacterial lipid production (Prema et al., 2015). - **Competition:** Bateria faces competition from well-established lipid sources (Prema et al., 2015).

The use of fish oils such as long-chain fatty acids (VLCFAs) may be safe for consumption at the established no observed adverse effect level (NOAEL), the transient lipid accumulation and changes in liver enzymes warrant ongoing monitoring and further studies to fully understand the long-term implications of their use (Tobin et al., 2024). In addition, Concerns about contaminants in marine animal co-products necessitate robust quality control measures, including hazard analysis and critical control point (HACCP) protocols, to ensure the safety and purity of lipid extracts (Monteiro et al., 2024). With advancements in purification techniques and eco-friendly sourcing, marine-derived lipids hold significant potential as a high-quality, bioactive option for lipid nanoparticles, enhancing the effectiveness of drug and gene delivery.

##### 2.5.1.2 Insects and bugs

Insects are recognized as a rich source of protein, fats, and essential fatty acids, making them an excellent candidate for inclusion in food products. Insects like *Hermetia illucens* (Black Soldier Fly), mealworms (*Tenebrio molitor*), and crickets (*Acheta domesticus*) are particularly notable for their lipid content. These lipids include both unsaturated and saturated fatty acids, with some species containing high amounts of linoleic acid (omega-6 fatty acid) and oleic acid (omega-9 fatty acid), which are beneficial for human health.

The use of insect lipids in food has a dual advantage of providing nutritional benefits while also reducing the environmental impact typically associated with traditional livestock production. As insects require less land, water, and food compared to conventional animals, their farming contributes to a more sustainable food production system. In addition, insects have the potential to convert organic waste into high-value proteins and fats, further enhancing the circular economy.

Insect lipids, especially those from *Hermetia illucens*, have gained attention for their application in sustainable food products. These lipids can be incorporated into energy bars, snacks, and even functional foods designed to support human health. The rich profile of essential fatty acids in insect lipids can improve the nutritional quality of food, providing omega-3 and omega-6 fatty acids that are often lacking in modern diets. Moreover, their ability to enhance the texture and mouthfeel of food products, such as baked goods and spreads, adds an additional layer of utility.

A growing trend in the food industry is the use of insect-derived lipids in vegan or plant-based alternatives, as they can offer a sustainable substitute for traditional animal fats used in such products. Since insect lipids are highly digestible and have favorable organoleptic properties (taste, aroma, texture), they can help mimic the sensory qualities of animal-derived fats, making them appealing for a wider range of consumers.

Although insect-derived lipids show potential for use in formulating lipid nanoparticles (LNPs) for drug delivery, research into this specific application is still limited. On the other hand, insect-derived lipids are gaining traction in the cosmetics industry due to their fatty acid profiles that aid in skin regeneration and wound healing. In particular, chitin and chitosan, sourced from insect exoskeletons, are utilized in wound dressings for their antibacterial properties and tissue-repairing abilities (Franco et al., 2021a). These lipids are commonly extracted using methods like Solvent Extraction, Supercritical Fluid Extraction (SFE), Pressurized Liquid Extraction (PLE), and Enzymatic Extraction (Franco et al., 2021a).

##### 2.5.1.3 Mammals and their waste

In comprehensive study of fatty acid compositions in major organs of mice, rats, rabbits, pig, horses, oxs, and sheep found that fatty acid patterns of neutral lipid fractions possess animal specificity (Veerkamp et al., 1962). This means that they differ from animal to animal but resemble one another for different tissues of one animal (Veerkamp et al., 1962). Another study studied the fat content of the *biceps femoris* and *longissimus dorsi* muscles of different animal spices under different environmental conditions. They found that the lipid content of muscles is significantly different. For example, game animals have higher levels of polyunsaturated acids, such as 18:2, 18:3, and 20:4, compared to ranges or feedlot steers. Additionally, game animals have higher levels of 16:1 and lower levels of 18:1 compared to range steers, while the muscles of range steers contain less of all fatty acids except 18:3 when compared to those of feedlot steers (Miller et al., 1986). This difference is attributed to the higher levels of phospholipids in game animals, which contribute to the higher levels of polyunsaturated acids in their muscle triglycerides (Miller et al., 1986).

Waste animal fats (WAFs) were utilized in one study for lipids extracted from leather fleshing, which is primarily subcutaneous fat, and from slaughterhouse waste, which is mainly intramuscular fat (Srinivasan et al., n.d.). Although the paper discussed its utilization in biodiesel production, the source can be utilized for other applications such as LNP production. One paper discusses the utilization of slaughterhouse by-products, specifically focusing on the extraction and analysis of fats and oils in meat and meat products (Khan et al., 2021b). Another paper attempted to increase the level of fatty acid composition in muscle and adipose tissue in beef cattle by feeding the cattle linseed or fish oil, this increased the levels of n-3 polyunsaturated fatty acids (PUFA) in the meat (Scollan et al., 2001) (Table.1). There are risk factors associated with this source and much is to be considered when utilizing it for lipid yield. The raw material has to go through treatments to remove all contaminants prior to extracting oils (Ali Ijaz Malik et al., 2024). Those treatments include filtration and sometimes bleaching to reduce impurities and ensure lipid safety (Haedrich et al., 2020). One of the risk factors associated with such sources of lipids is the contamination with Bovine spongiform encephalopathy (BSE) (Seidel et al., 2006). This risk can be reduced and the prions can be degraded and inactivated through hydrolytic fat splitting (Müller et al., 2006). However, sampling and testing post treatment is imperative to ensure safety. Additionally, the slaughterhouses of which you would acquire the animal fat waste have poor sanitation and are associated with a number of pathogens (Ovuru et al., 2024). The by-products of animal waste, including fat, are documented to be susceptible to microbial contamination (Jayathilakan et al., 2012). The contamination concerns are not limited to the biological, pesticides have been documented to accumulate in animal tissues and fats (Gilbert, 2005). Although the degree and concentration of the environmental contamination may differ based on the location, the chronic exposure is expected to accumulate contaminants in animal fat waste (Kamarianos et al., 2003). Therefore additional steps such as distillation for purity and glycerolysis for refining lipids aid in risk reduction (Ndiaye et al., 2020).

Lipid peroxidation, especially in polyunsaturated fatty acids (PUFAs), can produce reactive compounds that degrade the quality of LNPs (Valgimigli, 2023). During harvesting, the microsomal and mitochondrial enzymes will initiate the process that ends with changes to the chemical structure of the lipids (Dragoev, 2024).One of the main product of this process is reactive carbonyl compounds, and the accumulation of these product increased the risk of cognitive functions impairment (Wang et al., 2024). In addition, routine testing and controlled processing conditions of animal products where processing should occur are temperature-controlled conditions to minimize the enzymatic activity that can initiate lipid degradation (Giordano et al., 2024).

The use of mammalian waste for lipid nanoparticle (LNP) production presents a unique opportunity for sustainable and efficient resource utilization in pharmaceuticals. By tapping into waste products such as tallow, bone marrow, and other lipid-rich byproducts, LNP manufacturing can benefit from a steady, cost-effective lipid source that might otherwise be discarded. Since mammalian lipids often share structural similarities with human lipids, they may improve compatibility and effectiveness for drug delivery applications.

#### 2.5.2 Animal derived

##### 2.5.2.1 Milk

Milk is a rich source of lipids, which are essential for various biological functions in the body. There are several methods for extracting lipids from milk, including centrifugation, solvent extraction, enzyme-assisted extraction, and membrane filtration (add references). In one study, the lipid content was extracted from two different milk samples using the Gottlieb-Röse method (Galanos and Kapoulas, 1965). The lipid content was found to be 2320 mg per 100 ml, while for another sample, it was significantly higher at 3360 mg per 100 ml (Galanos and Kapoulas, 1965). This variation highlights the natural variability in milk lipid content, which can be influenced by factors such as the diet of the dairy animals, their breed, and the method of milk processing and handling (Galanos and Kapoulas, 1965). In another study, the source of milk lipid secretion was heavily investigated. The source of lipids in milk originated from the endoplasmic reticulum (ER) of the mammary gland cells and was found to be mainly made of triacylglycerols (Mather and Keenan, 1998). They are synthesized in or on the surfaces of rough ER membranes and are released into the cytoplasm as microlipid droplets (MLDs) with a surface coat of proteins and polar lipids (Mather and Keenan, 1998). Then MLDs may fuse with each other to form larger cytoplasmic lipid droplets (CLDs) that are eventually secreted from the cell (Mather and Keenan, 1998). A study focused on human milk found that the types of lipids in milk are primarily triglycerides, which account for the majority of milk lipids (McManaman, 2009). Additionally, the cytoplasmic lipid droplets (CLDs), which are precursors of milk lipids, consist of a hydrophobic core of neutral lipids, including triacylglycerol (TAG) as well as cholesteryl and retinyl esters (McManaman, 2009; MacGibbon, 2020). Other studies suggest that LNPs are even naturally present in breast milk, however these liposomes were not investigated for any applications (Srinivasan et al., n.d.; Khan et al., 2021b).

Bovine milk has present as microscopic globules in an oil-in-water emulsion, serving primarily to provide energy to the newborn calf (Gordon, 2013). The fat content in bovine milk can vary significantly, ranging from about 3.0 % to 6.0 %, which is influenced by factors such as the breed of cow, diet, and stage of lactation (Gordon, 2013). Another study found that the main types of lipids found in milk are triacylglycerols, which comprise more than 98 % of bovine milk lipids (German and Dillard, 2006). Other fatty acids present in bovine milk include diacylglycerols, monoacylglycerols, and phospholipids in smaller quantities (German and Dillard, 2006). Another paper confirmed that along with triacylglycerols, phospholipids and cholesterol are the main lipid constituents of the amphipathic fat globule surface membrane exposed to the watery milk fraction. Additionally, human milk lipids provide essential lipid-soluble vitamins and polyunsaturated fatty acids (PUFAs), including long-chain polyunsaturated fatty acids (LC-PUFAs) such as linoleic acid (LA), α-linolenic acid (ALA), arachidonic acid (AA), eicosapentaenoic acid (EPA), and docosahexaenoic acid (DHA) (Koletzko et al., 2001).

Milk is not only a source of lipids, but it has also been proven to have crucial therapeutic benefits as it has been discussed in a study that used full-fat goat milk products (Woods et al., 2022). The milk contained 26 % total fat and 13 % milk fat in dry matter, which had a similar effect on disease progression (Woods et al., 2022). In contrast, skim goat milk powder, which lacks fat content, showed no detectable activity in mitigating the disease (Woods et al., 2022). The composition of milk lipids and various bioactive substances present in milk fat have proven to provide multiple benefits (Dhankhar et al., 2016). The benefits may include anti-cancer properties, cholesterol-lowering effects, and hormone synthesis support due to the presence of bioactive lipids such as conjugated linoleic acid (CLA), trans fatty acids (TFA), phospholipids, ether lipids, and sterols (Dhankhar et al., 2016). This is further evident in its potential therapeutic applications, as milk polar lipids have been associated with various physiological benefits across all age groups, including effects on brain and immune function, and lipid metabolism (Venkat et al., 2024). The bioactive components derived from milk lipids, such as phospholipids and sphingolipids, have been associated with various health-promoting effects, including cardiovascular health, immune function, and potentially anti-carcinogenic properties (Venkat et al., 2024). It has been documented to possess antimicrobial properties, particularly free fatty acids and monoglycerides, for their potential to protect infants from infections (Isaacs and Thormar, 1991).

In pharmaceuticals, using milk lipids could enhance the safety and efficacy of LNPs, as they may reduce immunogenicity and improve compatibility with the human body. Furthermore, milk-based lipids are already widely used in supplements and food products, which makes the regulatory pathway for certain applications potentially more accessible. With advancements in milk fractionation and purification technologies, the future of milk lipids in LNPs is promising.

##### 2.5.2.2 Egg yolk

The lipid content of egg yolk may differ based on environmental factors, including dietary intake of fatty acids by hens (Sirri and Meluzzi, 2011). It can impact the synthesis of arachidonic acid (AA), which plays a significant role in the lipid composition of egg yolks, primarily as a product synthesized from linoleic acid (Sirri and Meluzzi, 2011). It was shown that when hens' diets are enriched with sources of n-3 polyunsaturated fatty acids (PUFAs), such as fish oil or algae, the synthesis of AA decreases (Sirri and Meluzzi, 2011). It can also be affected by high-pressure and thermal treatments that can alter the structures and functionalities of egg yolk components, such as their solubility, rheological behavior, aggregation, and emulsifying properties (Anton, 2013). The composition of egg yolk extracts significantly influences the mechanical properties, stability, and structure of derived liposomes (Kondratowicz et al., 2019). Carotenoids and tocopherols, play critical roles in determining liposome characteristics, with carotenoids generally enhancing stability and tocopherols showing mixed effects (Kondratowicz et al., 2019).

The general lipid profile of egg yolk is primarily 65 % of the dry matter (Xiao et al., 2020). This includes 62 % triglycerides, 33 % phospholipids, and less than 5 % cholesterol (Xiao et al., 2020). The edible parts of egg yolk also contain proteins, mainly in the form of lipoproteins, which include high-density lipoproteins (HDLs), low-density lipoproteins (LDLs), and very low-density lipoproteins (VLDLs) (Xiao et al., 2020). Using high-performance liquid chromatography (HPLC), a study was able to identify plasmalogen as a unique lipid in egg yolk (Palacios and Wang, 2005). It is a unique class of phospholipids characterized by a vinyl-ether bond at the sn-1 position of the glycerol backbone and an ester bond at the sn-2 position (Palacios and Wang, 2005). Egg yolk contains a wide diversity of essential lipids that serve structural roles in membranes and function in cell signaling (Wood et al., 2021). It also includes polyunsaturated fatty acids in various structural lipid families, which are important for generating pro-resolving mediators involved in anti-inflammatory and anti-proliferative pathways (Wood et al., 2021).

Egg yolk lipids, particularly phospholipids (PLs), are extensively used in the pharmaceutical industry due to their emulsifying properties (Huopalahti et al., 2007). They are employed in parenteral lipid emulsions and drug delivery systems, including liposome and fluorocarbon emulsions (Huopalahti et al., 2007). Egg yolk has become the preferred source of lecithin for these applications (Huopalahti et al., 2007). Egg yolk-derived lipids can be used to synthesize LNPs for different applications, such as drug delivery (Tang et al., 2020a). They showed efficient delivery of therapeutic agents, low toxicity, and good stability in mouse models (Tang et al., 2020a). One study found that alpha-tocopherol content, the ratio of polar and nonpolar acylglycerols (AG), and the composition of phospholipids were identified as crucial parameters influencing the permeability of liposomal membranes formed from various egg yolk extracts (Kondratowicz et al., 2018). Egg yolk-derived lipids, with their long-standing use in vaccine delivery systems, present valuable potential for advancing drug and gene delivery technologies in the future.

#### 2.5.3 Human source

Most of the lipids extracted from human sources were mainly to study the lipids themselves, these sources can be from human skeletal muscle tissue and other human tissue (Kumar Srivastava et al., 2006). One study used LDL particles isolated from pooled plasma of healthy volunteers to identify over 350 molecular lipid species in 19 lipid subclasses and provides insights into the roles and localization of predominant lipid classes in LDL (Reis et al., 2013). Screening these lipids can reveal their properties, which can be utilized for their characteristics, such as in a paper that discusses the antimicrobial properties of sphingolipids, fatty acids, and other lipids isolated from different parts of the human body (Fischer, 2020).

In studying the lipids isolated from a human source in the field of drug delivery, exosomes seems to be the main interest of researchers (Pegtel and Gould, 2019). Exosomes are small vesicles released by cells that play crucial roles in intercellular communication (Pegtel and Gould, 2019). They are involved in various physiological and pathological processes by transferring proteins, RNA, and other molecules between cells (Pegtel and Gould, 2019). The lipid composition of exosomes is crucial for their formation, release, and biological functions (Skotland et al., 2019). They contain specific lipid classes like cholesterol, sphingomyelin, phosphatidylcholine, phosphatidylserine, and phosphatidylethanolamine, with variations in lipid content from different cell types suggesting heterogeneity in their composition (Skotland et al., 2019). MSC-derived exosomes have exhibited the ability to enhance in vitro mesenchymal stromal cell chondrogenesis and hypertrophy, indicating their role in tissue engineering and regeneration (Deng et al., 2018). Exosomes have also been employed in cancer studies to understand the crosstalk between adipocytes and liver cancer cells and the bioactive molecules that influence their behavior, growth, metastasis, and immune responses (Rios-Colon et al., 2020).

Exosomes, or microvesicles (MV), are produced and secreted by both tumor and normal cells, and they carry a variety of molecular signatures that reflect the nature of their cells of origin (Wieckowski and Whiteside, 2006). For example, tumor-derived MV expresses death ligands such as FasL and TRAIL, which mediate the apoptosis of activated T cells, contributing to tumor escape mechanisms (Wieckowski and Whiteside, 2006). On the other hand, dendritic cell (DC)-derived MV are characterized by the presence of co-stimulatory molecules and HLA class I and II antigens, which play roles in regulating immune responses (Wieckowski and Whiteside, 2006). Despite its therapeutic possibilities, it is essential to consider the cell of origin as it may result in unwanted outcomes (Mrizak et al., 2015). For example, nasopharyngeal carcinoma (NPC)-derived exosomes, whose interaction with Treg plays a role in immune evasion, tumor progression, and immune regulation in NPC (Mrizak et al., 2015). Exosomes derived from the mesothelioma cell line, which expressed the highest levels of NKG2D, have been found to down-regulate the expression of NKG2D, an important receptor for immune cell activation, leading to impaired immune responses against tumors (Clayton et al., 2008).

As it has been exhibited in the literature, a cell of origin can be a double-edged sword. Multiple studies screened different types of cells of origin, such as exosomes derived from human umbilical cord mesenchymal stem cells (HUCMSC-EXOs), in various medical conditions (Yaghoubi et al., 2019). Studies found that HUCMSC-EXOs may reduce inflammation, promote tissue regeneration, improve cognitive function, and enhance insulin sensitivity in these conditions (Yaghoubi et al., 2019). NK cell-derived exosomes express NK cell markers and killer proteins, exhibit cytotoxic activity against tumor cells and activated immune cells, and are taken up by tumor cells (Lugini et al., 2012). Another documented therapeutic potential was that of fibrocyte-derived exosomes in promoting angiogenesis and accelerating wound closure in diabetics (Geiger et al., 2015). These exosomes were able to enhance endothelial cell proliferation, new vessel formation, and re-epithelialization, along with their cargo of proteins and microRNAs that activate key processes in wound healing (Geiger et al., 2015). Despite the promising application in dermatology, the application is limited due to several factors, one of which is the exosome isolation cost (Bai et al., 2024). Recently, exosomes derived from HEK293 cell line were compared to soybean extract-based product called SeleMix-AT™ and found that the HEK293 cell line derived exosomes has similar benefits in cell proliferation, anti-inflammatory effects, and protection from skin irritation (Kim et al., 2024). In another application, HEK293 cell line derived exosomes were loaded with miR-365a-3p mimics and successfully delivered the miRNA to HepG2 cells, increasing apoptosis, offering a potential therapeutic strategy for hepatocellular carcinoma (HCC) (Ghotaslou et al., 2024). While the therapeutic potential of exosomes is widely documented, The safety of the human derived exosomes remains a challenge, as there isn't an established standard of evaluation (Hu et al., 2024). In addition, cell derived exosomes aren't necessarily always biocompatible or less toxic (Herrmann et al., 2021). It's important to note that exomes are not FDA approved, and it's adverse effects are not yet fully understood (Bai et al., 2024). With the first clinical trial published in august 2024 (Akhlaghpasand et al., 2024).

Using human-derived lipids for lipid nanoparticles (LNPs) has intriguing potential, especially for personalized or autologous therapies, as they are biocompatible and offer unique molecular characteristics specific to human biology. They could advance targeted drug delivery and therapeutic applications, particularly in regenerative medicine and immune response modulation.

#### 2.5.4 Plant source

Plant-derived lipids encompass a wide variety of compounds, including fatty acids, wax lipids, and isoprenoids (Jansen and Wiesenberg, 2017). Plants may exhibit lipids with different properties and compositions, which can be influenced by both genetic factors and environmental conditions (Table 1) (Jansen and Wiesenberg, 2017). For instance, differences in fatty acid composition have been observed between temperate C3 and C4 crops and even within the same species (Jansen and Wiesenberg, 2017). Such as different genotypes of kale and swede variations in the chain length distribution of wax lipids, indicating genetic control through variation in the enzyme system (Jansen and Wiesenberg, 2017). Plant-derived lipids offer several unique advantages due to their natural origin and composition, as they are non-toxic and have low immunogenicity, making them safer for clinical applications compared to synthetic alternatives (Sarvarian et al., 2022). They have several advantages such as low toxicity and immunogenicity, the ability to be easily modified for specific targeting, the protection they offer to various substances from the external environment, and their potential for large-scale, eco-friendly production of lipid nanoparticles when compared to animal source (Tinnirello et al., 2023).

The plant lipid composition contributed to the effectiveness of the NLCs by providing a lipid with a lower melting point by using lipids such as oleic acid, and similarly to SLNs by using solid lipids such as tripalmitin (Figueiredo et al., 2022). The type of plant can contribute to the properties of the extracted lipid, as is the case with lipids derived from ginger, specifically phosphatidic acid (PA). PA was shown to inhibit the growth of *P. gingivalis*, a pathogen associated with periodontitis, at very low concentrations (Sundaram et al., 2019). The term “edible” has been used when referring to plant lipid sources such as grapefruit and ginger, which means they are recognized and processed by the human body in a way that is similar to food intake (Yang et al., 2018). The advantages of plant-derived edible nanoparticles being edible include their potential to serve as natural therapeutics against a variety of diseases due to their ability to mediate cell-cell communication similarly to mammalian exosomes (Zhang et al., 2016; Gai et al., 2024). It was recorded in the literature that nanovesicles obtained from grapefruit, termed Grape fruit derived nanoparticles (GFDNs), exhibited notably high levels of phosphatidylcholine (PC, 29 %) and phosphatidylethanolamine (PE, 46 %) within their composition (Yang, 2021; Wang et al., 2014). Plant-derived exosome-like nanoparticles (PENs) and edible plant-derived nanoparticles (epNPs) show promise for disease management and drug delivery (McClements, 2013). These nanoparticles, sourced from plants like corn, citrus, grapefruit, ginger, and broccoli, offer improved solubility, stability, permeability, bioavailability, reduced side effects, a longer half-life, and targeted tissue delivery compared to traditional carriers (Bollineni et al., 2024). Research investigating ginseng-derived nanoparticles (GSDNs) using mass spectrometry found that they consist of DGMG (59.4 %), PE (16.8 %), and ceramide (13.8 %). Notably, DGMG and ceramide are unfamiliar lipid components in other plant-derived nanoparticles, distinguishing the lipid composition of GSDNs from those of similar origins (Cao et al., 2019).

Another study presents findings indicating that ginger-derived extracellular lipid nanoparticles (GELNs), demonstrate more potent pharmacological effects compared to extracellular lipid nanoparticles derived from fruits or vegetables (Zhu and He, 2023). GELNs exhibited notable antioxidant and anti-inflammatory effects on macrophages, whereas extracellular lipid nanoparticles derived from grapes, grapefruit, and carrots did not display similar effects (Zhu and He, 2023). A documented study investigates how exosome-like nanoparticles from edible plants, termed EPDENs, affect mammalian cells (Mu et al., 2014). Results from EPDEN-transfected macrophages reveal that ginger EPDENs prompt increased expression of antioxidation-related genes like heme oxygenase-1 and the anti-inflammatory cytokine IL-10, while EPDENs from grapefruit, ginger, and carrot promote the activation of nuclear factor-like (erythroid-derived 2) (Mu et al., 2014).

The use of plant-derived lipids for lipid nanoparticles (LNPs) holds strong future potential due to their natural abundance, sustainability, and ability to be tailored for specific applications. As extraction techniques and lipid engineering improve, plants can offer a scalable, eco-friendly alternative for high-quality LNPs in pharmaceuticals and other industries. This shift could reduce dependency on animal sources and synthetic lipids, supporting a more sustainable approach to LNP production.

#### 2.5.5 Microorganisms source

Rising demands due to population growth and climate change highlight the need for alternative lipid sources. Microbial lipid production, influenced by genetic and environmental factors, offers advantages over plants and marine oils (Table.1). It provides cost-effective, customizable structures through genetic modification and enables sustainable, pesticide-free production year-round in a smaller footprint (Ghazani and Marangoni, 2022). Microbial lipids are utilized for its several advantages, including food packaging applications, as they are considered promising alternatives to petro-based polymers due to their sustainability, bio-renewability, biodegradability, and environmentally benign nature (Zubair et al., 2021). In addition, there is a growing demand for cocoa butter alternatives due to supply shortages and the high cost of exotic fats like illipe butter and mango fat (Patel et al., 2020). Microbial lipids can mimic the lipid composition of cocoa butter, making them suitable for use in chocolate production and other food products that require specific fat properties (Patel et al., 2020). The production of polyunsaturated fatty acids (PUFAs) by microorganisms like fungi and microalgae can reduce production costs by valorizing waste materials and offering environmental benefits through waste detoxification (Kothri et al., 2020). The use of waste material addresses the cost of the carbon source, which can account for as much as 80 % of the production expenses (Kothri et al., 2020). Additionally, the production process itself requires further refinement to enhance lipid efficiency and productivity such as various culture modes, genetic engineering techniques, and the valorization of by-products, which is a common practice in vegetable oil processing (Szczepańska et al., 2022). The microorganism requires lipids for a multitude of its metabolic functions, such as communication and energy storage, as well as structural supports of the cell membrane (Subramaniam et al., 2010). Although the synthesis of lipids is natural, the accumulation of these lipid products is often attributed to Oleaginous microbes (Subramaniam et al., 2010). When extracting lipids from microorganisms for medical applications, several safety considerations must be taken into account. First, and in the case of algal biomass the potential for contamination with heavy metals and other pollutants is a significant concern, as it can accumulate harmful substances from their environment  (Dai et al., 2022). Additionally, the extraction processes often involve solvents, some of which may be toxic or environmentally harmful (Santoro et al., 2019; Zapata-Boada et al., 2022). As the extraction solvents are similar in all microorganisms, this is true for all the microorganisms mentioned in this section. Despite oleaginous microbes being generally recognized as safe (GRAS) (Alhattab et al., 2024). understanding the biological activity and potential side effects of the extracted lipids is essential (Ryan et al., 2010). Otherwise, they offer advantages like year-round production in small spaces, biodegradability, and the potential to valorize waste materials, especially in producing polyunsaturated fatty acids (PUFAs). With rising population demands and environmental concerns, microbial lipid production is becoming an increasingly attractive alternative to traditional lipid sources.

##### 2.5.5.1 Yeast

Oleaginous yeasts can accumulate 40–70 % of their dry weight in lipids, with high diversity in those lipid profiles attributed to different yeast species (Ratledge, 2004) (Table.1). Yeast can be highly efficient in lipid production, with certain strains like *Lipomyces starkeyi* being specifically highlighted for their capability to produce microbial oils efficiently from various substrates, including crude glycerol (Caporusso et al., 2021). Lipid production from *Yarrowia lipolytica* by metabolizing sustainable substrates, such as volatile fatty acids (VFAs) has demonstrated a high potential for biofuel production. However, the processing costs limit its application and industrial commercialization (Naveira-Pazos et al., 2023). A paper exploring the lipid yield, productivity, and fatty acid profiles of different oleaginous yeast strains found that *C. oleaginosus* demonstrated the highest biomass and lipid yields (Shaigani et al., 2021). They reported about 7.5 g/L lipids in wheat straw hydrolysates and 3.8 g/L lipids in brown algae hydrolysates with versatile substrate utilization, productivity, and tolerance towards fermentation inhibitors in complex media (Shaigani et al., 2021). Another study compared the fatty acid compositions of the lipids produced by the two yeast strains (i.e., *C. podzolicus* DSM 27192 and *T. porosum* DSM 27194) (Qian et al., 2020). Lipids were mainly made of carbon chain lengths of C16 and C18, the high content of C18:1 in *C. podzolicus* DSM 27192 is particularly favorable for its positive impact on the cold flow properties of biodiesel (Qian et al., 2020). The fatty acid profile of the single-cell oil (SCO) produced by *R. toruloides* strains showed a predominance of oleic acid (C18:1) ranging from 50.3 to 63.4 %, followed by palmitic acid (C16:0) with percentages between 23.9 and 31.0 % (Michou et al., 2022). This indicates a high content of monounsaturated fatty acids (MUFAs) and a significant presence of polyunsaturated fatty acids (PUFAs), albeit in lower percentages (Michou et al., 2022). The fatty acid composition can be affected by the culture medium, as observed in one study of microbial oils produced from waste fish oil which contained high amounts of omega-3 (PUFAs) (Fabiszewska et al., 2021). In contrast, microbial oil extracted from yeast cells grown in olive oil medium (MO5) showed a significant decrease in the content of unsaturated oleic acid from 76 % to about 50 %, and an increase in the content of saturated acids, especially palmitic acid, to a value exceeding 20 % of all fatty acids (Fabiszewska et al., 2021).

*Rhodosporidium toruloides* is considered a promising chassis organism for lipid production and biotechnological applications due to its diverse substrate appetites, robust stress resistance, and other favorable features (Wen et al., 2020). *Cutaneotrichosporon oleaginosus* is regarded as a versatile strain in the context of lipid production due to its ability to utilize a wide range of substrates, including volatile fatty acids, seagrass waste hydrolysate, waste-activated sludge, and aromatics (Tang et al., 2020b). *Candida viswanathii* Y-E4 can achieve a higher lipid yield (13.6 g/L, corresponding to 0.08 g of lipid/h) when grown on crude glycerol compared to other yeast strains (Guerfali et al., 2020). It can exceed yeasts grown in controlled bioreactors such as *R. diobovatum* (6.9 g/L), *R. graminis* (10.3 g/L), *T. spathulata* JU4–57 (5.0 g/L), and *Sporidiobolus pararoseus* KM281507 (3.26 g/L), highlighting its potential for economic production of microbial oils from biodiesel-derived crude glycerol (Guerfali et al., 2020). Yeast-derived lipids present a sustainable, scalable, and customizable alternative for lipid nanoparticles (LNPs), offering the potential for large-scale, cost-effective production and a more ethical option compared to animal-based lipids.

##### 2.5.5.2 Algae

Lipids synthesized by microalgae fall into two main categories: polar and non-polar lipids, also known as neutral lipids (Stoyneva-Gärtner et al., 2022). Polar lipids play vital roles in cell structure and signaling, commonly referred to as structural or membrane lipids (Stoyneva-Gärtner et al., 2022). Despite constituting of a small portion (about 20 %) of the total lipid content in cells, they typically consist of extractable fatty acids (FtAs) with long chains (Stoyneva-Gärtner et al., 2022). Non-polar lipids serve various biological purposes, with triglycerides often highlighted for their role in energy storage (Stoyneva-Gärtner et al., 2022). A study Explored lipid-based nanosystems developed from *Nannochloropsis* sp. algae lipids, serving as carriers for lipophilic antioxidants like curcumin and tocopherol, by analyzing both loaded and unloaded LNPs (Clemente et al., 2021). These LNPs showed some acceptable structural characteristics, localization, and functional interplay crucial for potential drug delivery (Clemente et al., 2021). The findings of this paper emphasized the active role of cargo molecules in shaping the aggregate structure, revealing diverse coexisting mesophases, with a prominent cubic symmetry structure (Clemente et al., 2021).

The lipids produced from *Chromochloris zofingiensis* are considered superior to plant oils in many ways and are viewed as the next-generation biofuel feedstock (Tsarpali et al., 2021; Kou et al., 2020). It can synthesize substantial amounts of lipids under photoautotrophic, heterotrophic, and mixotrophic modes, and co-produce high-value carotenoids like astaxanthin along with triacylglycerol, making it a promising candidate for biofuel production (Kou et al., 2020). Seaweed lipids have been identified as promising phytochemicals with intrinsic bioactive properties, including antioxidant, antitumor, anti-inflammatory, and antimicrobial activities (Lopes et al., 2021). These lipids have some potential applications across various industries including pharmaceutical, nutraceutical, and cosmeceutical fields due to their health-promoting effects (Lopes et al., 2021). *Chlorella vulgaris* is recognized for its high lipid content and productivity, making it an efficient strain for lipid production due to its ability to grow under various conditions, which further contributes to its efficiency in lipid production (Soleimani Khorramdashti et al., 2021).

As it was the case with utilizing yeast in lipid production through raw waste material, the approach was applied to algae. In addition to using sludge, *Chlorella pyrenoidosa* lipid production was significantly enhanced through mixotrophic culture conditions, especially with the addition of optimal volatile organic acids (VOAs), increasing biomass production by 1.9 to 2.4 times compared to the blank culture (Tan et al., 2020). Despite the challenges posed by high initial capital and production costs, the opportunities microalgae present as sustainable biofactories include their rapid growth rates, non-competition with arable land, and the potential for wastewater utilization, which together with biorefinery approaches, could improve the economic feasibility of microalgae cultivation (Fernandes and Cordeiro, 2021).

##### 2.5.5.3 Bacteria

Oleaginous bacterial species are favored for their high cell growth rates as well as its ability to accumulate up to 70 % of their dry weight in lipids (Dong et al., 2016). This synthesis of lipids takes place in the stationary phase (Dong et al., 2016). *Rhodococcus opacus*, *Arthrobacter* sp., and *Acinetobacter calcoaceticus*, are capable of storing components of fatty acids up to 87 % of their cell dry weight (CDW) and can generate large biomass in a short period due to high growth rates (Kumar et al., 2020). *Bifidobacterium* extracted polysaccharides exhibit diverse structures, including exopolysaccharides (EPS) and cell-wall polysaccharides (CWP), found in strains like *B. pseudocatenulatum, B. longum*, and others (Pyclik et al., 2020). Polar lipids in *Bifidobacterium* species vary in phospholipid and glycolipid content and function; for instance, *B. longum* spp. infantis ATCC 15702 displays specific lipid fractions and glycolipids affecting immune responses (Pyclik et al., 2020). The lipid accumulation efficacy of bacteria generally shows low lipid accumulation compared to other microorganisms (Koreti et al., 2022). They are produced in tiny droplets within the cytosol, and some strains can accumulate oil under specific environmental conditions, with polyhydroxyalkanoic acids being the most abundant class of neutral lipids (Koreti et al., 2022). Triacylglycerol accumulation has been reported in some cases, mainly occurring during the stationary growth phase of bacterial culture (Koreti et al., 2022). *Acinetobacter* is one of the known oleaginous bacterial strains in lipid production (Salcedo-Vite et al., 2019). It can produce lipids from multiple carbon sources, such as glucose, pyruvate, ethanol, and acetate, and can do so in high concentrations (Salcedo-Vite et al., 2019). Recently, a genetic engineering study was conducted to alter the cell morphology of *Acinetobacter baylyi* ADP1, by changing the cell morphology from rod-shaped to spherical, which allowed for increased accumulation of intercellular lipids (Luo et al., 2022).

*Escherichia coli* has been proposed as a promising fatty acid producer. The overexpression of Acetyl-CoA carboxylase can exhibit an increase in fatty acid synthesis which allows the utilization of *Escherichia coli* as a fast-growing producer of lipids (Meng et al., 2011). To improve production of fatty acid in *Escherichia coli*, one study analyzed the relation between lowering the abundance of the membrane protein ompF and an increase in fatty acid tolerance and fatty acid production (Tan et al., 2017). Another study assessed *Pseudomonas aeruginosa's* ability to produce lipids and found a broad spectrum of fatty acids with different chain lengths (de Andrés et al., 1991). It has been found that *Pseudomonas aeruginosa* possesses the ability to produce rhamnolipids, which are structured by the link between one or two rhamnoses to an equivalent number of fatty acids (i.e. saturated or unsaturated) (Haba et al., 2003). One study employed *Pseudomonas aeruginosa* to produce biofuel from waste papers after 72 h of incubation, utilizing the metabolic pathways to efficiently produce biofuels (Prema et al., 2015).

The future potential of bacteria-derived lipids for lipid nanoparticles (LNPs) is highly promising, especially as we seek sustainable and efficient lipid sources for large-scale production. Bacteria can be genetically engineered to produce tailored lipids with properties optimized for LNP stability, cellular uptake, and targeted release. With advancements in synthetic biology, bacterial systems could provide precise control over lipid composition, enabling customization for specific drug and gene delivery applications. Furthermore, bacterial lipids offer a cost-effective and eco-friendly alternative to animal-based sources, supporting scalability in nanomedicine manufacturing.

### 2.6 Challenges of lipid sources

The challenges faced in lipid sourcing include the structural diversity of lipids, which complicates analysis due to the various combinations of polar headgroups, fatty acyl chains, and backbone structures (Bou Khalil et al., 2010). In addition, the lack of a single uniform analytical platform for the analysis of all lipids presents a significant challenge, including all potential enantiomeric, stereoisomeric, and regioisomeric lipid species that can be identified (Bou Khalil et al., 2010). Lipid sourcing involves issues related to API loading, stability, polymorphic transitions, and manufacturing complexities (Corzo et al., 2020) (Table 1). Furthermore, complete lipid extraction is a difficult process due to the complex nature of biological samples (Pati et al., 2016). Since it often requires optimization of sample preparation techniques to overcome challenges related to extraction efficiency and the complete removal of non-lipid contents (Pati et al., 2016).

The solubility of poorly water-soluble drugs in triglyceride lipids, particularly long-chain triglycerides, which is often low, can limit the effectiveness of lipid-based formulations in enhancing the bioavailability of these drugs (Porter et al., 2007). Additionally, the fatty acid composition of the triacylglycerols, especially 16:0 and 18:1 fatty acids, can vary significantly, affecting the physical properties of animal-based samples such as milk fat (Logan and Lutcavage, 2008). The unambiguous identification of molecular species (qualitative analysis) as well as precise quantitation remain significant challenges as the availability of reference standard material as well as relevant internal standards continues to be limited, which complicates both the identification and quantitation processes (Murphy, 2018). The complexity of the chemical composition of lipids presents a fundamental challenge in biology, which presents challenges for their availability and precise sourcing for specific applications (Harayama and Riezman, 2018). As the composition of lipids play a role in determining the proper admission route of LNP's, for example, the mobility of LNP's in a mucus environment depends on PEG-lipid density (Ongun et al., 2024). The properties determined by the composition of lipids are also vital to the successful drug delivery, in oral admissions, the presence of lipids increases absorption of the loaded drug (Mehrdadi, 2024). In addition, when using lipid injectable emulsions stabilizing agents are required such as egg yolk phospholipids and sodium oleate to maintain a homogenous distribution of lipid droplets and prevent destabilizing processes like aggregation or coalescence (Driscoll and Bistrian, 2024). In Table 2 we summrize the composition and admission route relevance to drug delivery.

**Table 2:** The aspects of relations of lipid composition to the selection of admission routes.

	Parenteral Lipids	Oral Lipids
The Absorption Process	Administered directly into the bloodstream, delivering drugs that have poor stability and absorption in the gastrointestinal tract (Driscoll and Bistrian, 2024; Sharma et al., 2023).	The administered oral lipids are Digested in the gastrointestinal tract, broken down by bile salts and enzymes before absorption (Yang et al., 2018).
Stability Requirements	The administration route requires sterility and stability to avoid aggregation or embolisms. Therefore, emulsifiers like egg yolk phospholipids or sodium oleate are often used to maintain stability (Driscoll and Bistrian, 2024).	When lipids encapsulate drugs that are administered orally, stability increases due to the reduction of chemical and enzymatic drug degradation (Mehrdadi, 2024; Mohite et al., 2023).
Formulation	These lipids require specific emulsifiers to prevent fat globule formation which ensure stability in the bloodstream (Otero-Millán et al., 2024).Additionally, the oxidation and hydrolysis of drugs sensitive to the administration route can be reduced by formulating parenteral lipids with superfine oils (Wilkinson et al., 2022).	The formulation of these types of Orally administered lipids can include a wider range of lipids, with plant based ones being a promising source (Jansen and Wiesenberg, 2017). Generally, the drugs are dissolved in a compatible lipids (or a blend of different lipids), with compatibility determined by the chemical nature of the drug (Holm et al., 2023).
Size of Lipid Particles	Nanoemulsions or small lipid droplets size plays an important role in its bioavailability, aggregation and concentration in the bloodstream (Chavda et al., 2024). Furthermore, the size of the lipid particle plays a crucial role in its successful reach to challenging target sites (Zirpoli et al., 2024).	There are varying sizes recorded in literature depending on the lipid source, with no issues regarding aggregation so long as they are stable and well-dispersed. (Zhang et al., 2016)
Common Lipid Sources	Common sources to obtain parenteral lipids are fish oils and soybeans oil (Gulotta et al., 2014; Raman et al., 2017).	There is a broad range of dietary fats including saturated fats, mono- and polyunsaturated fats from various plant and animal sources that can be used to formulate these oral lipids. (Jansen and Wiesenberg, 2017; Figueiredo et al., 2022; Franco et al., 2021b)
Safety and Regulatory Standards	High regulation standards are required to ensure the infusion safety (Driscoll, 2006). As well as the possibility of infection and Hyperlipidemia (Raman et al., 2017).	There are Fewer stringent sterility requirements, but it must meet general safety standards (Chen, 2008).

In micro-algal communities, optimizing lipid production for biofuel applications remains a challenge (Stockenreiter et al., 2012). While technical and genetic engineering modifications have been extensively explored for yield enhancement, the ecological dynamics of algal diversity present a less investigated but potentially cost-effective avenue for improving lipid yields (Stockenreiter et al., 2012).

Lipids derived from human samples may present a complex and large number of lipid species, which makes the analytical analysis of samples challenging (Quehenberger et al., 2010). This is further compounded by the fact that lipids, unlike gene sequences or proteins, have not been as amenable to detailed analysis with the modern tools of genomics and bioinformatics (German, 2011). Therefore, there is a critical need for mechanistic research to understand the composition, structures, and functions of lipids (German, 2011). In addition, the diversity of lipid chemical structures presents difficulties both from experimental and informatics perspectives making it hard to establish a globally accepted classification system (Fahy et al., 2011). There is a need to create a database of lipid structures and related genes/proteins to efficiently analyze experimental data, and manage metadata and protocols (Fahy et al., 2011). With recent advances in artificial intelligence (AI), AI may be used to integrate experimental data with existing knowledge into metabolic and signaling pathways and develop informatics software for efficient searching, display, and analysis of lipidomic data. The production of lipids, especially those derived from animal sources or monoculture crops like palm oil, can have significant environmental impacts that include deforestation, loss of biodiversity, and increased greenhouse gas emissions (Robert et al., 2020). The growing concerns about the sustainability of lipid sources encourage the search for more environmentally friendly and practical lipid sources. Lipids possess a wide variety of compositions, which is attributed to their source.

### 2.7 Conclusion

Lipid nanoparticles have emerged as a promising approach for delivering various therapeutic compounds, including small molecules and nucleic acids. However, the availability of suitable lipid sources for nanoparticle production remains a challenge. One potential source of lipids for nanoparticle production is natural resources. This review dives into the adoption of natural reservoirs in the supply of lipids for the creation of LNPs, illustrating a terrain where environmentally friendly and biocompatible sources act as foundational elements for LNP synthesis. Natural lipid reservoirs have surfaced as promising reservoirs for generating LNPs. In addition to lipid sources, there are also challenges associated with the production of lipid nanoparticles. However, researchers have been exploring various strategies to overcome these challenges via different methods and compositions. Some of these strategies involve the use of lipid conjugation techniques to improve drug loading, as well as the optimization of formulation manufacturing parameters. Although there are limitations and challenges in these technical approaches, there are emerging new approaches to combat these limitations.

Future research may also bring forth *bio-inspired LNPs*, mimicking natural structures for enhanced biocompatibility and stability, and *personalized LNPs*, optimized based on patient-specific genetic or cellular profiles, paving the way for precision medicine.

## CRediT authorship contribution statement

**Alanood S. Alfutaimani:** Writing – original draft, Software, Resources, Investigation, Data curation. **Nouf K. Alharbi:** Writing – original draft, Validation, Resources. **Amirah S. Alahmari:** Writing – review & editing, Supervision. **Almaha A. Alqabbani:** Writing – review & editing. **Abdulaziz M. Aldayel:** Writing – review & editing, Validation, Supervision, Resources, Project administration, Funding acquisition, Conceptualization.

## Declaration of competing interest

The authors declare the following financial interests/personal relationships which may be considered as potential competing interests.

Abdulaziz Mohammed Aldayel reports financial support was provided by Saudi Arabia Research Development and Innovation Authority. If there are other authors, they declare that they have no known competing financial interests or personal relationships that could have appeared to influence the work reported in this paper.

## Data Availability

No data was used for the research described in the article.
